# Unintended Consequences of Invasive Predator Control in an Australian Forest: Overabundant Wallabies and Vegetation Change

**DOI:** 10.1371/journal.pone.0069087

**Published:** 2013-08-21

**Authors:** Nick Dexter, Matt Hudson, Stuart James, Christopher MacGregor, David B. Lindenmayer

**Affiliations:** 1 Booderee National Park, Jervis Bay, Australia; 2 Fenner School of Environment and Society, National Environmental Research Program, The Australian National University, Canberra, Australia; University of Pretoria, South Africa

## Abstract

Over-abundance of native herbivores is a problem in many forests worldwide. The abundance of native macropod wallabies is extremely high at Booderee National Park (BNP) in south-eastern Australia. This has occurred because of the reduction of exotic predators through an intensive baiting program, coupled with the absence of other predators. The high density of wallabies at BNP may be inhibiting the recruitment of many plant species following fire-induced recruitment events. We experimentally examined the post-fire response of a range of plant species to browsing by wallabies in a forest heavily infested with the invasive species, bitou bush *Chrysanthemoides monilifera*. We recorded the abundance and size of a range of plant species in 18 unfenced (browsed) and 16 fenced (unbrowsed) plots. We found the abundance and size of bitou bush was suppressed in browsed plots compared to unbrowsed plots. Regenerating seedlings of the canopy or middle storey tree species *Eucalyptus pilularis*, *Acacia implexa*, *Allocasuarina littoralis*, *Breynia oblongifolia* and *Banksia integrifolia* were either smaller or fewer in number in grazed plots than treatment plots as were the vines *Kennedia rubicunda*, *Glycine tabacina* and *Glycine clandestina*. In contrast, the understorey fern, *Pteridium esculentum* increased in abundance in the browsed plots relative to unbrowsed plots probably because of reduced competition with more palatable angiosperms. Twelve months after plots were installed the community structure of the browsed and unbrowsed plots was significantly different (P = 0.023, Global R = 0.091). The relative abundance of *C. monilifera* and *P. esculentum* contributed most to the differences. We discuss the possible development of a low diversity bracken fern parkland in Booderee National Park through a trophic cascade, similar to that caused by overabundant deer in the northern hemisphere. We also discuss its implications for broad scale fox control in southern Australian forests.

## Introduction

The problem of overabundant herbivores changing ecosystem structure through grazing or browsing is a well recognised worldwide phenomenon in terrestrial and marine ecosystems [1] [2] [3] [4] [5] [6] [7]. For example, this is occurring in the hardwood forest ecosystems of much of Europe and North America, where the removal of predators and restrictions on hunting by humans has resulted in a substantial increase in deer populations leading to major impacts on forest structure and biodiversity [2], [4]. These heavily browsed temperate forest communities become *fern parkland* comprised of mature and senescent trees with a floristically depauperate understorey dominated by unpalatable ferns [8], [9], [10]. The impacts of persistent heavy browsing can extend through trophic cascades to influence not only plants but also invertebrates [11], birds [12], and soil micro-organisms [13].

In the *Eucalyptus* forests of southern Australia, relatively little attention has been paid to the ecological role of browsing mammals in shaping forest structure and floristics. Instead, wildfire has been considered to be the main form of disturbance influencing the composition and structure of these forests [14], [15], [16]. However, studies by Leigh and Holgate [17] and Parsons et al. [18] examined the response of a range of plant species to native mammal herbivory, (principally macropods: kangaroos and wallabies) in forest and woodland habitats following fire in southern Australia. Both these studies stressed that following fire, herbivores had a substantial effect on the recruitment of a wide range of native plants. Parallel to these investigations are studies in southern Australia on the impact of browsing macropods on the regeneration of commercially valuable *Eucalyptus* species after forestry operations [19], [20] and revegetation attempts during mine site rehabilitation [21], [22]. This research has revealed that mammalian browsing has major impacts on plant regeneration and plant species composition. These studies, together with literature on the impact of browsing ungulates in northern hemisphere forests [23], [24], suggest that herbivory may be an under-recognised factor influencing the composition and structure in Australian forests.

Despite their probable importance in shaping the structure of Australian forests, many small browsing macropods have suffered significant population decline, or even extinction due to predation by the introduced red fox (*Vulpes vulpes*) [24], [25] [26]. Indeed, a small forest macropod, the Tasmanian pademelon (*Thylogale billardierii*), has probably been extirpated from mainland Australia by fox predation. Yet it is a major pest in wood production forests in Tasmania where foxes are extremely rare [19]. Many fox control programs have been initiated in southern Australia in an attempt to reverse macropod decline [24], [25], [26], [27]. In most instances, these control programs have resulted in a large-scale increase in the abundance of terrestrial mammals, including wallabies [24], [25], [26], [27], [28]. This increase is evident even in large macropods such as eastern grey kangaroos *Macropus giganteus* [29].

Since 1999, Booderee National Park (BNP) on the south-east coast of Australia, has maintained an intensive fox control program that has resulted in the increase in abundance of a number of native mammal species [31], [32]. There has been an increase in the abundance of macropods of approximately 10 fold between 2003 and 2009. The swamp wallaby (*Wallabia bicolor)* is a specialist browsing macropod [33] and is the most common macropod species at BNP. It is also an important component of the diet of foxes in BNP [34], [35]. Less common mammalian herbivores present at BNP are the eastern grey kangaroo (*Macropus giganteus*), red necked wallaby (*Macropus rufogriseus*), common brushtail possum (*Trichosurus vulpecula*), common ringtail possum (*Pseudocheirus peregrinus*) and bush rat (*Rattus fuscipes*) [31]. European rabbits (*Oryctolagus cuniculus*) are also present but their distribution is very patchy and their numbers very low and were never observed on the study site. Coincident with the decrease in fox abundance and the increase in wallaby abundance, observations of the flora at BNP suggest that few seedlings have established after fire and that revegetation programs following fire have failed with most individual plants experiencing mortality from browsing.

**Figure 1:**
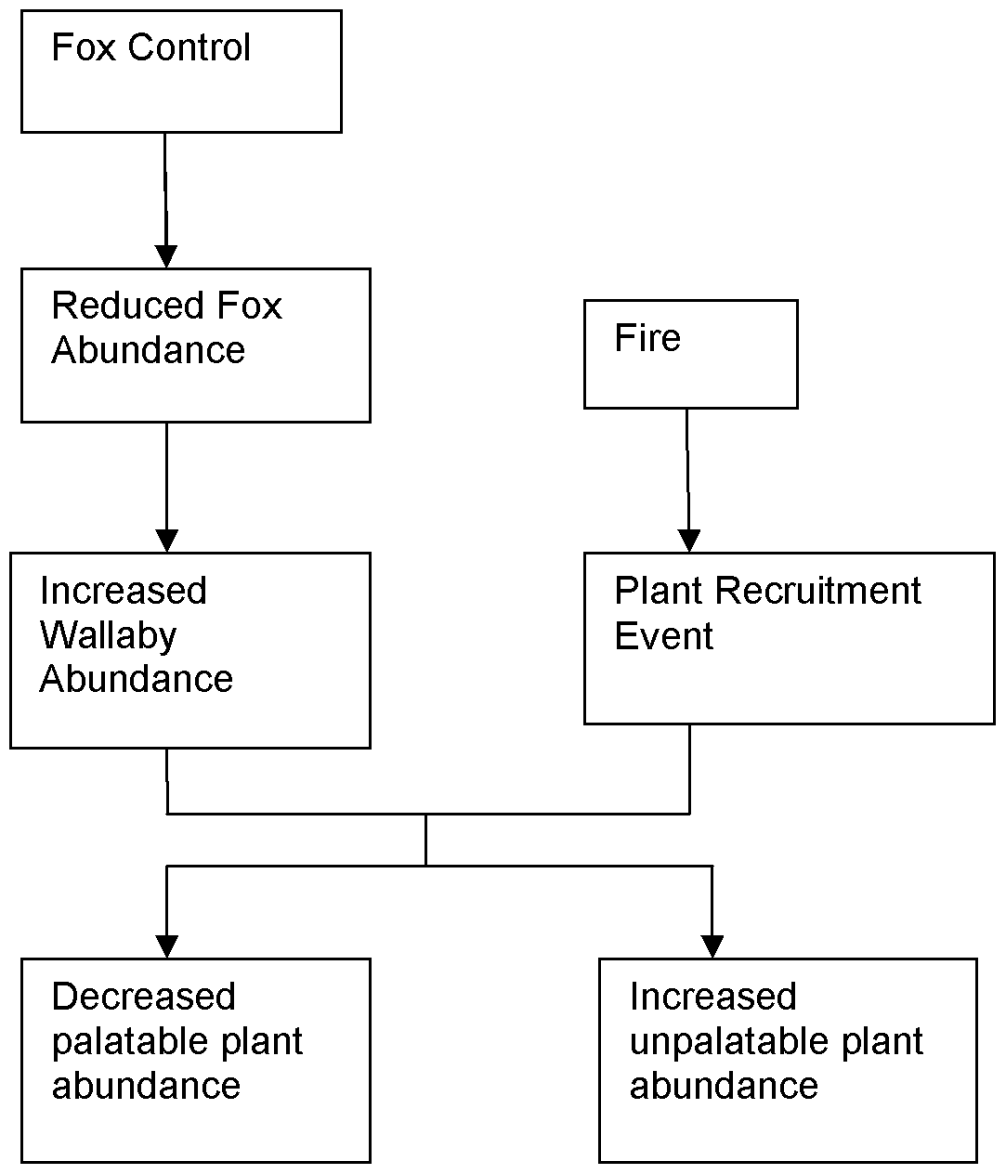
Conceptual model of impact of wallaby browsing on flora of Booderee National Park.

In this paper, we developed a new conceptual model (Figure 1) of possible long-term changes in the vegetation at BNP whereby the intensive control of foxes has led to a substantial increase in the abundance of wallabies. In turn, this will negatively affect the recruitment of palatable plant species following fire. We also predicted that the recruitment of unpalatable plants will be enhanced through reduced competition with more palatable species. Therefore, we designed an exclusion experiment to determine whether browsing by macropods was reducing the abundance and size of palatable plants and increasing the abundance and size of unpalatable plants. The browsing exclusion experiment was conducted over one year, following a burn to control the environmental weed; bitou bush, *Chrysanthemoides monilifera rotundifolia* in a mixed blackbutt *Eucalyptus pilularis*, dry sclerophyll forest. Forests dominated by *E. pilularis* are common throughout coastal eastern Australia [36].

Importantly, our study differs from earlier investigations of the impact of browsing herbivores where workers assumed that their study systems were in equilibrium. By contrast, in this study, we know that herbivore abundance is increasing and that the system was possibly shifting to a new equilibrium. Therefore, we wished to be able to make robust predictions about the possible future state of forested ecosystems at BNP and more broadly for other forested areas where fox control is being undertaken.

## Materials and Methods

We conducted this study at Booderee National Park, a national park in south-eastern Australia approximately 6500 ha in area (approximate midpoint is 35°10′S latitude, 150°40′E longitude). The area has a temperate maritime climate with an average rainfall of 1225 mm per annum spread relatively evenly over the year. BNP is unusual in having a high diversity of habitats ranging from rainforest to sedgeland in a relatively small area [37], [31].

Vegetation communities at BNP have been mapped and classified in detail by Taws [38]. Approximately one third of BNP has been invaded by *C. monilifera*. *Chrysanthemoides monilifera* is a perennial evergreen shrub in the family Asteraceae (a native of South Africa), and major environmental weed of dune and coastal forest in southern Australia [39]. At BNP a range of habitats is affected by *C. monilifera* invasion, but this invasive plant is most common in coastal dune shrubland formerly dominated by *Acacia sophorae* and *Leptospermum laevigatum* where *C. monilifera* was planted in the 1960s to stabilise the unstable dune system [40]. From the dune shrubland, *C. monilifera* invaded *E. botryoides* and *E. pilularis* dry sclerophyll forest adjacent to the dune system. In these communities, *C. monilifera* has formed a dense 2 to 3 m high monoculture. Aerial spraying of *C. monilifera* bush commenced in the late 1990s with the study site sprayed in June 2005 and partially resprayed in May–June 2006 prior to burning.

Our conceptual model of the hypothesised species responses is shown in Figure 1. We predicted that following the control burn, there would be proportionally more palatable plants in unbrowsed plots and that there would be proportionally more unpalatable plants in the browsed plots. However, predicting the response to browsing of plants that have different life forms using simple traits is difficult [41]. Nonetheless, based on life history characteristics and data in the literature on the susceptibility of different plant genera to grazing or browsing, we made predictions about the impact of herbivory on the abundance and size of 10 of the more common species.

For the four most common tree species, we predicted that seedlings or ramets of blackbutt *Eucalyptus pilularis*, coastal banksia *Banksia integrifolia*, lightwood *Acacia implexa* and black she-oak *Allocasuarina littoralis* were likely to be more abundant and taller in unbrowsed than browsed plots given the known susceptibility of these genera to browsing [42], [43], [44], [19], [45]. We also included coffee bush *Breynia oblongifolia* in the analysis as our initial observations indicated a heavy level of grazing pressure on this mid-storey tree. Scurr et al [46] demonstrated that the distribution of boneseed *Chrysanthemoides monilifera* ssp. *monilifera* in Tasmania was constrained by domestic and wild browsers. In light of this, we predicted the conspecific bitou *C. monilifera* ssp *rotundata* would also be less abundant and smaller in size within browsed plots. Some plants in the family Fabaceae have relatively high concentrations of nitrogen making them attractive to herbivores [47]. Therefore, we predicted that the twining legumes *Kennedia rubicunda* and *Glycine* spp would be heavily browsed and more abundant and larger in treatment plots than the controls [48].

In contrast, for two species (eastern nightshade *Solanum pungetium* and bracken fern *Pteridium esculentum*) we predicted that there would be an increase in size and abundance in browsed plots. For the highly prickly *S. pungetium*, we predicted an increase in size or abundance, as prickliness in Australian *Solanum* may be an adaptation to deter wallaby browsing [49]. As a dense understorey of unpalatable ferns is a common outcome of intensive browsing in northern hemisphere forests, we predicted an increase in the abundance and/or size of *P. esculentum. Pteridium esculentum* is relatively toxic and unpalatable to mammalian herbivores including macropods [50] due to a cyanogenic glucoside [51], [52].

Finally, we included two very common species in our analysis which respond rapidly to fire or disturbance; blady grass *Imperata cylindrica* and black nightshade *Solanum nigrum*. *Imperata cylindrica* is a tough rhizatomous grass that tends to become dominant with frequent burning [53] and *S. nigrum* a cosmopolitan ruderal, annual, weed [54]. We made no specific predictions about these two species as data on their palatability and toxicity are equivocal.

In August 2007, we established 18 unbrowsed plots (excluding macropod browsing) and 18 browsed plots (allowing macropod browsing). Unbrowsed plots consisted of a 3×3 m enclosure of 2.5 cm mesh wire 150 cm high, with the wire supported by strained metal star pickets at each corner. To access a fenced plot, we installed a ∼60 cm wide door consisting of a secured mesh flap. The wire mesh was buried to a depth of 20 cm to prevent access by burrowing mammals. Browsed plots were 3×3 m with star pickets on each corner but no mesh. Plots were not chosen at random but selected to maximise the probability that they would be burnt (i.e. high mass of dead *C. monilifera* canes). An effort was made to pair browsed and unbrowsed plots by separating them by at least 20 m. However, because of the heterogeneous dispersion of fuel sufficient to sustain a fire, not all plots could be paired. Therefore, the pairs of plots were not treated as blocks in the sense of a randomized block design experiment. It was assumed that both species of possums and bush rats would be able to access the unbrowsed plots.

The study sites were located in an area of forest that was part of a spray-burn-spray treatment for *C. monilifera*. This treatment involves spraying *C. monilifera* with the herbicide (glyphosate), allowing the plants to die and cure, and then burning the cured canes. This stimulates a mass germination by *C. monilifera* which can significantly reduce the seedbank. Before these seedlings can flower and set seed, the infestation is re-sprayed [55], [56]. An earlier study has indicated that recruitment of native plants was better following a spray-burn-spray treatment than either spraying alone, or spraying and burning [57].

Test burns indicated that when conditions were suitable for ignition of the cured canes, combustion of clumps was briefly intense for 5–10 minutes. Burning was conducted over two days in October 2007. We burnt a total of 226 ha. Combustion was rapid and relatively intense with flame heights up to 5 metres in tall (2–3 m) stands of cured *C. monilifera*. All 18 browsed sites were completely burnt, but two of the 18 unbrowsed sites were not burnt and were not subsequently used in the analysis reported here.

We began bi-annual fox control in 1999 using lethal 1080 baits placed at 1-km intervals along a network of trails throughout BNP (for a full description of the methods see Dexter et al [30]. Foxes were controlled as part of a long term program to protect biodiversity in the Park. The control of foxes using lethal 1080 baits is a required action under the Booderee National Park Plan of Management (1999): “5.10.10 Maintain a control program for foxes and monitor the program's effectiveness, amending control methods as required [58].” The Booderee National Park Plan of Management is the legal frame work that the Australian Federal Government (through Parks Australia) uses to manage the Park. As such an ethics approval is not required to control foxes.

Baiting was increased to monthly in 2003. Baits were checked after 14 days and the number of baits taken by foxes expressed as a percentage of the total number of baits available. Bait take is a rough index of abundance suitable only for measuring large scale changes in abundance over large areas but it has been used successfully to monitor changes in fox relative abundance in other studies [59] [30]

We counted macropods by repeated spotlighting along 109 permanent 100 m long transects established throughout BNP (for a full description of the methods see Lindenmayer et al [31]). Counts were not undertaken on nights of poor weather. Experienced observers completed the spotlighting surveys annually from 2003 to 2007, and biennially from 2007. Macropods were usually detected by sound of their footfall when they were disturbed. Where possible, the species of macropod was identified as swamp wallaby, eastern grey kangaroo or red necked wallaby. Wallabies were monitored under the Australian National University Animal Ethics Protocol number C.RE.60.09.″

One month after the fire, all the plots were surveyed. The following attributes were recorded; the number of individuals, percentage cover (in five classes <5%; 5–25%, 25–50%; 50–75%; 75–100%), height range (tallest and shortest individual for all species), and diameter range (widest and narrowest individual for species wider than tall) for all seedling and resprouting species. Mature trees were not included in this study. These surveys were repeated at two monthly intervals until November 2008 giving a total of seven repeated surveys.

Plant abundance data were the counts of the 12 individual species in each plot. The hypothesis that browsing affected species abundance was tested using the mixed models option with the diagonal offset covariance structure for the repeated-measure in the statistical program SPSS. Treatment (browsed, unbrowsed) was entered as a fixed factor, with sample session (1–7) entered as a repeated-measure and sampling session by treatment entered as an interaction term. Five species were not detected until the second sampling session (*A. littoralis*, *B.integrifolia*, *K. rubicunda*, *S. nigrum*, and, *S. pungetium*). For these species, sessions 2–7 were entered as the repeated-measure. For species with a highly right-skewed abundance distribution, data were transformed by the fourth root. Mixed models are better at handling the more complex covariance structure values than traditional repeated measure ANOVAs, including missing values [60]. They are also relatively robust to non-normality [61].

For the analysis of plant size, the median height in centimetres for nine of the 12 species, recorded in both unbrowsed and browsed plots were also entered into a mixed model. For *Glycine spp*, *K. rubicunda* and *S. pungetium* the median width in cm was used as this dimension tends to be greater than height for these species.

To determine whether macropod browsing influenced the overall community structure of the regenerating vegetation, we compared the differences between browsed and unbrowsed plots using an analysis of similarity (ANOSIM). We used the November 2008 data for plant abundance for all species detected as these represented the final and most diverse community sample. The ANOSIM was used after square root transforming the data to emphasise the importance of rarer species. To determine which species contributed most strongly to differences between browsed and unbrowsed plots, we entered all the transformed data into a Similarity Percentage Analysis (SIMPER). Both these analyses were completed using PRIMER v6 [62].

We extended the analysis of community structure to include an index of species mass. We did this by repeating the ANOSIM but using a composite value consisting of the number of each species in each plot in November 2008 multiplied by the median size (height or width). As with the ANOSIM for numbers of individuals, the data were square root transformed to emphasise the importance of rarer species. These data also were analysed with a SIMPER.

## Results

Fox abundance, as indexed by bait take, declined precipitously from 24% of baits taken in 1999 before starting to recover to 32% in 2003 when monthly baiting commenced [63]. This brought bait take to a consistently low level (less than 1% of baits taken) [63] However, it is unlikely that true abundance of foxes declined as much as the bait take, as foxes continue to be observed in low numbers.

Results from spotlight transect counts revealed a 10 fold increase in the abundance of wallabies. A total of 5 individuals were detected in 2003 compared to 60 in 2009 [63]. The majority of observations were of swamp wallabies (77.1% of observations), with fewer eastern grey kangaroos (19.4% of observations) and red-necked wallabies (3.5% of observations). Over the same period common brushtail possums increased while common ringtail possums declined [31]

**Figure 2:**
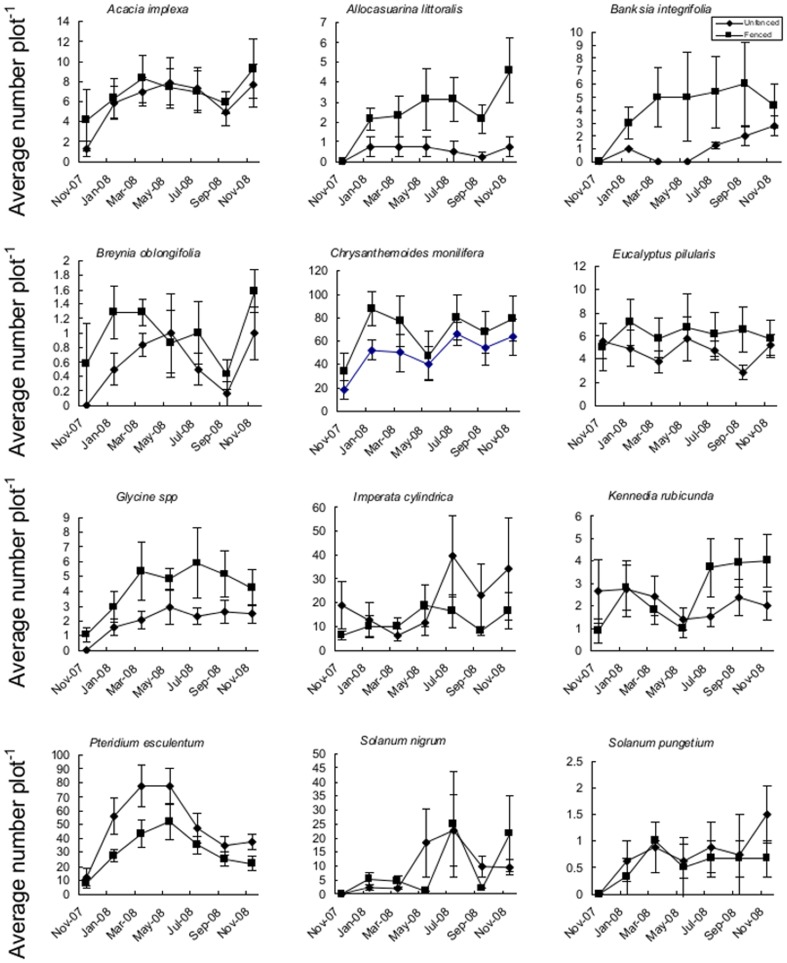
Mean numbers of 12 plant species in (3×3 m) browsed by macropods (n = 18) and (3×3 m) unbrowsed plots(n = 16) (±1SE) from Booderee National Park, south-eastern Australia.

Browsing significantly reduced the mean number of seedlings and resprouts for 6 of the 12 species surveyed (Table 1, Figure 2). The results accorded largely with our predictions about which species would be significantly affected by browsing; *Chrysanthemoides* (P = 0.038), *Eucalyptus* (P = 0.006), *Banksia* (P<.0001), *Allocasuarina* (P = 0.001), *Breynia* (P = 0.036) and *Glycine* (P = 0.001) were all significantly reduced in abundance in the browsed plots compared to the unbrowsed plots. However, the differences in abundance for *Eucalyptus* and *Banksia* had almost disappeared by the last count. The other main canopy species, *Acacia implexa*, was not significantly affected by macropod browsing. There were no significant interaction effects detected between treatment and sample session for any species examined.

**Table 1:** Mixed model results for numbers of 12 plant species in (3×3 m) browsed by macropods (n = 18) and (3×3 m) unbrowsed plots(n = 16) (±1SE) from Booderee National Park, south-eastern Australia.

Species	Factor	Numerator d.f.	Denominator d.f.*	F	Significance
*Acacia implexa*	Treatment	1	168.41	0.66	0.416
	Session	6	48.104	1.67	0.15
	Treatment x Session	6	48.104	0.136	0.991
*Allocasuarina littoralis*	Treatment	1	39.95	11.93	0.001
	Session	5	15.51	0.36	0.864
	Treatment x Session	5	15.51	0.285	0.914
*Banksia integrifolia*	Treatment	1	65.227	15.99	0.000
	Session	5	32.468	1.124	0.367
	Treatment x Session	5	32.468	0.752	0.590
*Breynia oblongifolia*	Treatment	1	4.63	4.631	0.036
	Session	6	4.12	4.117	0.006
	Treatment x Session	6	0.30	0.301	0.930
*Chrysanthemoides monilifera*	Treatment	1	199.35	4.367	0.038
	Session	6	53.729	3.246	0.089
	Treatment x Session	6	0.149		0.989
*Eucalyptus pilularis*	Treatment	1	118.213	86.14	0.006
	Session	6	45.278	6.209	0.000
	Treatment x Session	6	45.278	1.359	0.252
*Glycine spp*	Treatment	1	106.81	11.76	0.001
	Session	6	50.05	6.94	0.0001
	Treatment x Session	6	50.05	0.49	0.856
*Imperata cylindrica*	Treatment	1	53.48	1.23	0.27
	Session	6	22.41	1.80	0.146
	Treatment x Session	6	22.41	0.77	0.605
*Kennedia rubicunda*	Treatment	1	117.33	0.719	0.398
	Session	6	38.56	2.126	0.072
	Treatment x Session	6	38.56	1.40	0.239
*Solanum pungetium*	Treatment	1	42.78	0.421	0.52
	Session	5	16.34	0.33	0.89
	Treatment x Session	5	16.34	0.13	0.98
*Solanum nigrum*	Treatment	1	45.99	3.17	0.08
	Session	6	19.42	1.46	0.24
	Treatment x Session	6	19.42	1.68	0.36
*Pteridium esculentum*	Treatment	1	112.25	11.65	0.001
	Session	6	68.21	8.77	0.0001
	Treatment x Session	6	68.21	0.67	0.672

* Denominator degrees of freedom calculated using Welch-Satterthwaite Equation and are therefore not necessarily integers.

On average, the abundance of the prickly nightshade *Solanum pungetium* was higher in browsed plots (0.875 to 0.65) but was not significantly different between grazed and unbrowsed plots (P = 0.52). However, there were significantly more *P. esculentum* ramets in browsed plots than unbrowsed plots (P<0.001). The abundance of the rapidly recolonising species *I. cylindrica* and *S. nigrum* were not affected by browsing.

**Figure 3:**
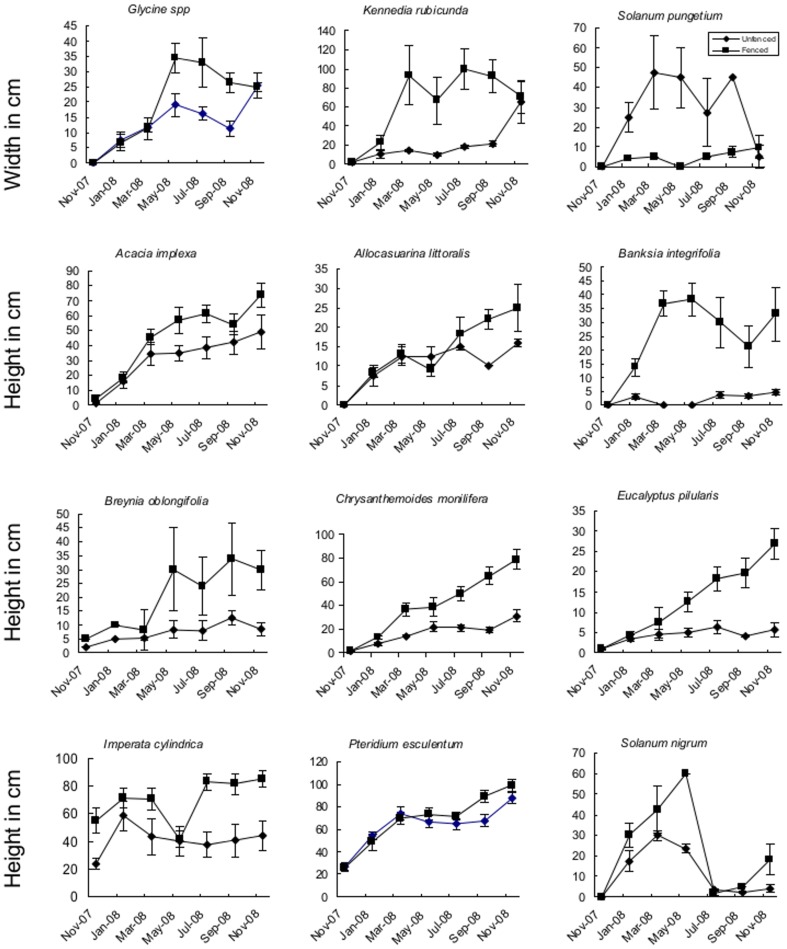
Mean size of 12 plant species in (3×3 m) browsed by macropods (n = 18) and (3×3 m) unbrowsed plots(n = 16) (±1SE) from Booderee National Park, south-eastern Australia.

The overall pattern was for larger plants to occur in unbrowsed plots compared to browsed plots. *C. monlifera* (P<0.0001), *E. pilularis* (P<0.0001), *A.implexa* (P<0.0001), and *B. Integrifolia* (P<0.0001) *S. nigrum*, (P<0.0001) and *I. cyclindrica* (P<0.001) were significantly taller in unbrowsed plots while *K. rubicunda* (P<0.0001) and *Glycine* (P = 0.003) were wider in unbrowsed plots (Table 2, Figure 3).

**Table 2:** Mixed model results for size of 12 plant species in (3×3 m) browsed by macropods (n = 18) and (3×3 m) unbrowsed plots(n = 16) (±1SE) from Booderee National Park, south-eastern Australia.

Species	Factor	Numerator d.f.	Denominator d.f*.	F	Significance
*Acacia implexa*	Treatment	1	124.03	14.62	0.0001
	Session	6	43.36	40.32	0.0001
	Treatment x Session	6	43.36	1.43	0.226
*Allocasuarina littoralis*	Treatment	1	17.47	1.46	0.224
	Session	5	7.08	2.75	0.109
	Treatment x Session	5	7.08	1.11	0.432
*Banksia integrifolia*	Treatment	1	24.55	23.84	0.0001
	Session	5	7.84	3.00	0.0001
	Treatment x Session	5	14.78	1.48	0.261
*Breynia oblongifolia*	Treatment	1	85.57	0.32	0.57
	Session	6	20.36	28.58	0.0001
	Treatment x Session	6	20.36	24.51	0.0001
*Chrysanthemoides monilifera*	Treatment	1	115.35	97.21	0.0001
	Session	6	44.49	99.05	0.0001
	Treatment x Session	6	44.49	19.98	0.0001
*Eucalyptus pilularis*	Treatment	1	90.88	32.88	0.0001
	Session	6	31.59	20.54	0.0001
	Treatment x Session	6	31.59	5.88	0.0001
*Glycine sp*	Treatment	1	16.79	11.58	0.003
	Session	6	7.83	187.3	0.0001
	Treatment x Session	5	8.48		
*Imperata cylindrica*	Treatment	1	77.44	41.88	0.0001
	Session	6	25.97	2.61	0.041
	Treatment x Session	6	25.97	0.95	0.479
*Kennedia rubicunda*	Treatment	1	24.92	40.04	0.0001
	Session	5	24.23	8.88	0.0001
	Treatment x Session	5	24.23	5.0	0.003
*Solanum nigrum*	Treatment	1	22.32	40.95	0.0001
	Session	5	11.60	42.84	0.0001
	Treatment x Session	5	11.60	28.17	0.0001
*Solanum pungetium*	Treatment	1	5.69	5.66	0.057
	Session	5	31.7	5.84	0.081
	Treatment x Session	5	3.25	8.73	0.045
*Pteridium esculentum*	Treatment	1	153.95	3.54	0.062
	Session	6	45.1	47.7	0.0001
	Treatment x Session	6	45.1	1.65	0.157

* Denominator degrees of freedom calculated using Welch-Satterthwaite Equation and are therefore not necessarily integers.

There were strong interactive effects between sample session and treatment for C. *monlifera* (P<0.0001), *E. pilularis* (P<0.0001), *A.implexa* (P<0.0001), and *B. Integrifolia* (P<0.0001) *S. nigrum*, (P<0.0001) and *I. cyclindrica* (P<0.001). The significant interactive effects indicated that in the unbrowsed plots plants continued to grow during the study but that in the browsed plots most plants remained stunted by browsing.

There was no significant treatment effect for *S. pungetium* but the interaction term between treatment and sample session was significant (P = 0.045). This interactive effect is shown in Figure 3 and indicates S. *pungetium* was much larger in browsed than unbrowsed plots for most of the study. However, by the end of the study, plants in unbrowsed plots had died and only small seedlings remained in browsed and unbrowsed plots.

A total of 70 species was detected during the vegetation surveys with 62 species recorded in the unbrowsed plots and 62 species detected in the browsed plots. Species detected only in browsed plots were *Centaurium erythraea*, *Ehrharta erecta*, *Eustrephus latifolius*, *Lagenifera stipitata*, *Persoonia linearis*, *Pratia purpurascens and Wahlenbergia gracilis*. These species tended to be small herbs or grasses (except *P. linearis and E.latifolius*). Similarly, the small (<10 cm) succulent forb *Crassula sieberana* was detected in only one unbrowsed plot and in six browsed plots. The species recorded only in unbrowsed plots were *Acacia ulicifolia*, *Billardiera scandens*, *Gahnia clarkei*, *Glochidion ferdinandi*, *Omolanthus populifolius*, *Pelargonium australe*, *Poa labillardieri*,and *Trema tomentosa*. Three of these species (*G*. *ferdinandi*, *O. populifolius* and *T. tomentosa*) were the seedlings of large shrubs or small trees that commonly form a mid-storey in coastal sclerophyll forest and rainforest.

**Figure 4:**
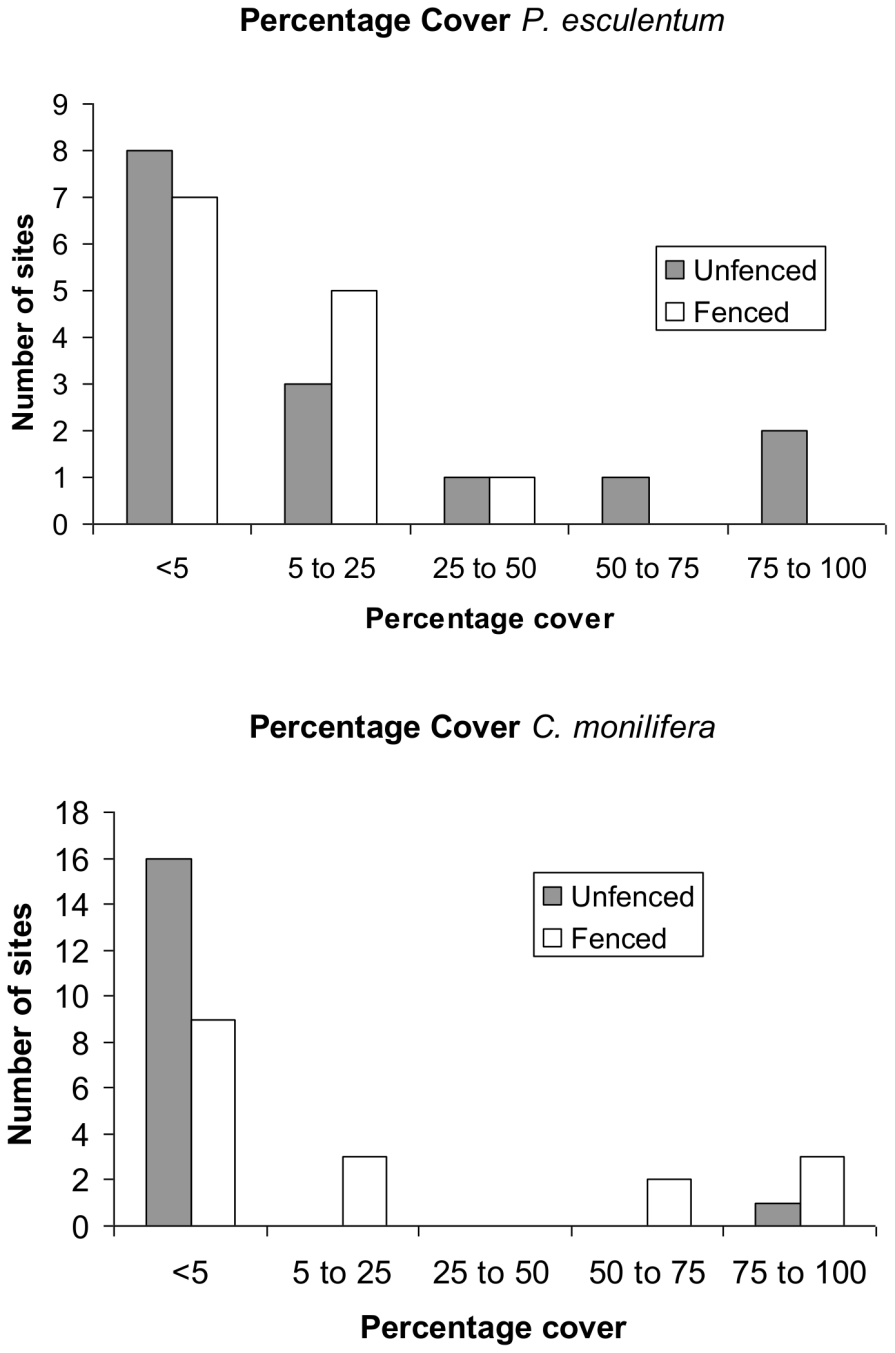
Percentage cover of *C. monilifera* and *P. esculentum* in browsed and unbrowsed plots.

The results of ANOSIM revealed significant differences in species composition between browsed and unbrowsed plots (Global R = 0.091, P = 0.023). The species contributing up to 50% of the dissimilarity between browsed and unbrowsed plots are listed in Table 3. The ANOSIM of species mass showed a larger difference between browsed and unbrowsed plots (Global R = 0.195. P<0.001) with species contributing up to 50% of dissimilarity between browsed and unbrowsed plots listed in Table 4. The contribution of the two most important species *C. monilifera* and *P. esculentum* to the observed differences in community structure can be seen by examining Figure 4 which shows the distribution of percentage cover classes of both species. It is clear that *P. esculentum* was more likely to be dominant in the unfenced plots than the fenced plots (Table 4).

**Table 3:** Similarity Percentage Analysis (SIMPER) of vegetation species abundance that contribute up to 50% of the variance between browsed and unbrowsed plots.

Species	Cumulative percentage
*Chrysanthemoides monilifera*	9.07
*Pteridium esculentum*	16.41
*Imperata cylindrica*	21.52
*Solanum nigrum*	25.66
*Conyza bonariensis*	29.77
*Acacia implexa*	33.79
*Entolasia marginata*	37.11
*Lomandra longifolia*	40.29
*Monotoca elliptica*	43.45
*Glycine sp*	46.54
*Oplismenus imbecillis*	49.22

**Table 4:** Similarity Percentage Analysis (SIMPER) of vegetation species mass that contribute up to 50% of the variance between browsed and unbrowsed plots.

Species	Cumulative percentage
*Chrysanthemoides monilifera*	15.91
*Pteridium esculentum*	27.07
*Acacia implexa*	33.70
*Imperata cylindrica*	40.17
*Entolasia marginata*	45.01
*Glycine sp*	48.48

## Discussion

Our study demonstrated that following fire at sites protected from browsing, a wide variety of palatable plants were more common or larger in unbrowsed sites than in browsed sites. Our study also demonstrated that two species of unpalatable plants benefited from the reduced competition with palatable plants in browsed sites. Of broader theoretical and practical importance, our study demonstrated the unforseen impacts on vegetation of a program to control an exotic carnivore. This pattern is similar to that seen on sub-Antarctic islands where feral cat *Felis catus* control led to an irrruption in exotic European rabbit *Oryctolagus cuniculus* numbers, and in turn, resulted in serious negative impacts on native vegetation [64].

Many Northern Hemisphere studies have been conducted in the ecological context of high deer densities that have existed for up to 50 years [66], [8], [10], hence, researchers were already examining heavily altered ecosystems. In Australia studies on the impacts of macropod browsing on managed forests and mine site revegetation predation by either foxes or dingoes is likely to have had a constraining effect on the abundance of macropods [23], [21], [22].

The lack of large mammalian predators is a novel circumstance in forests in eastern Australia.Prior to the introduction of foxes, wallabies would have been hunted by both Aboriginal people and dingoes (*Canis lupus dingo*) [65]. We therefore believe that at BNP, browsing pressure is now much higher than in the recent past, although the actual level of browsing pressure is unknown with certainty. The significance of our study is its timing at the beginning of a phase of very high herbivore abundance.

We have assumed that BNP is unusual because heavy browsing has resulted from intensive fox control. However, intensive fox control is becoming more common. Indeed, 100,000s of ha of forest in south-eastern and south-western Australia now under intensive fox control [27], [28]. Our experiment shows that an unexpected outcome of such control efforts may be major structural and floristic change to these forests due to increases in wallaby abundance.

We found that overstorey species (*E. pilularis*, *B. integrifolia*, *A. implexa* and *A. litoralis*) were either reduced in abundance, size, or both due to wallaby browsing. However, this difference may not carry through to maturity given processes such as self thinning, as this is very common in *Eucalyptus* species following fire [67]. The lack of significant interactive effects between sample session and treatment on abundance indicates that despite many plants being removed by browsing, differences in abundance remained consistent through the course of the study. This is because most species continued to germinate and resprout, thereby adding new recruits to both browsed and unbrowsed sites at roughly equal rates. Profound effects of macropod browsing on both *Banksia* and *Eucalyptus* regeneration have previously been documented [43], [68], [20]. Compared to understorey species, trees are buffered against death and damage from browsing by eventually growing higher than herbivores can browse [69]. However, some trees may become permanently stunted and remain within the browsing zone if browsing pressure is strong enough [68]. This may be the case for species like *B. integrifolia* and *E. pilularis*, which had significant interactive terms in the analysis indicating that while plants in unbrowsed plots continued to grow, plants in browsed plots hardly changed height over the entire course of the study (Figure 2). Despite the relative immunity of canopy trees to browsing, evidence from North America indicates that the composition of canopy hardwoods can be changed from more palatable species to less palatable species as a result of intense browsing pressure [2].

Palatable understorey species such as *B. oblongifolia*, *K. rubicunda* and *Glycine spp.* remain exposed to browsing throughout their life cycle and may be susceptible to extirpation [2]. *Kennedia spp*. have persistent seed banks that are stimulated to germinate by fire [70], so they can potentially survive long periods even if all adult plants are consumed by browsers. In contrast, *S. pungetium* was substantially larger in browsed plots than unbrowsed plots for most of the study. This may be because this sprawling unpalatable annual forb had reduced competition from other more palatable plants in the browsed plots. There was a tendency for some very small (<5 cm) grasses and forbs to be present or more abundant only in browsed plots. This is most likely due to a lack of larger more vigorous competitors. Shorter growing species tend to increase compared to taller growing species, under moderate grazing pressure, in structurally simple grasslands, although this trend may not hold at higher grazing pressure [71], [72].

The two species that contributed most to differences between browsed and unbrowsed plots were *C.monilifera* and *P. esculentum.* This occurred in both our analyses of abundance and mass. *Chrysanthemoides monilifera* dominated the unbrowsed plots while *P. esculentum* dominated the browsed plots. This is despite *P. esculentum* being a dietary item for swamp wallabies [73]. However, di Stefano and Newell [33] showed that bracken was not a preferred food of swamp wallabies. Subtle differences in phenolics within plant families [74] and within individual genera [75] can have profound impacts on the browsing pressure to which individual plant species are exposed. The substantial impact of browsing on *C. monilifera* is in accord with the finding of Scurr et al [46] that browsing by macropods and domestic livestock limited the distribution of the conspecific boneseed *Chrysanthemoides monilifera* ssp. *monilifera* in Tasmania. In our study, *C. monilifera* did not grow higher than 1 m in the browsed plots, compared to unbrowsed plots where it grew up to 2 m. Furthermore, in only one browsed plot did *C. monilifera* have a percentage cover of more than 5%. Hence, its ability to exert its strong competitive suppressive impact on regenerating native flora was greatly reduced by browsing, although it would still be able to suppress competition through the allelopathic properties of its roots [76].

*Pteridium esculentum* was more abundant in the browsed plots than unbrowsed plots, possibly because its toxicity not only discouraged browsing but also its allelopathy reduced competition from *C. monilifera* and the more diverse angiosperm flora in unbrowsed plots. The increased dominance of *P.esculentum* is consistent with northern hemisphere studies which demonstrate an increase in ferns with high levels of deer browsing [77], [10]. In Australia, *P. esculentum* has been shown to have a substantial negative competitive effect on eucalypt seedling survival [78], so there may be an additive negative effect on eucalypt survival in the browsed plots. In North American studies, ferns were shown to exacerbate the effect of deer browsing by limiting the germination of a range of angiosperms [79].

Our study points to a larger problem of macropod browsing beyond wood production forests and mine site regeneration in the management of Australian forests. In our study part of a naturally occurring community of plants was altered. This study suffers the limitations of other exclosure studies in that the treatment represents an extreme case of the complete exclusion of terrestrial browsers [80]. A further limitation maybe that increased macropod mortality during a large scale wildfire may mean less intensive post fire browsing. It is also limited to one particular ecosystem, and ecosystem infested with a major environmental weed. This may make inferences about more pristine forests difficult. However, our work does point the way for future studies of vegetation change where large scale exotic predator manipulation may be considered for environmental benefit.
